# Construction of a mortality risk prediction model for patients with acute diquat poisoning based on clinically accessible data

**DOI:** 10.1186/s12995-024-00416-7

**Published:** 2024-05-21

**Authors:** Mingxiu Lv, Yu Du

**Affiliations:** 1 West China School of Public Health and West China Fourth Hospital, Sichuan University, Chengdu, Sichuan China; 2 Department of Emergency and Critical Care Medicine, West China School of Public Health and West China Fourth Hospital, Sichuan University, Chengdu, 610041 Sichuan China; 3 Health Emergency Management Research Center, West China-PUMC C.C. Chen Institute of Health, Sichuan University, Chengdu, 610041 Sichuan China

**Keywords:** Diquat poisoning, Prediction model, Logistic, Nomogram, Influencing factors

## Abstract

**Background:**

To examine the risk factors associated with mortality in individuals suffering from acute diquat poisoning and to develop an effective prediction model using clinical data.

**Methods:**

A retrospective review was conducted on the clinical records of 107 individuals who were hospitalized for acute diquat poisoning at a tertiary hospital in Sichuan Province between January 2017 and September 30, 2023, and further categorized into survivor and nonsurvivor groups based on their mortality status within 30 days of poisoning. The patient’s demographic information, symptoms within 24 h of admission, and details of the initial clinical ancillary examination, as well as the APACHE II score, were documented. The model was developed using backward stepwise logistic regression, and its performance was assessed using receiver operating characteristic curves, calibration curves, Brier scores, decision curve analysis curves, and bootstrap replicates for internal validation.

**Results:**

Multifactorial logistic regression analysis revealed that blood pressure (hypertension, OR 19.73, 95% CI 5.71–68.16; hypotension, OR 61.38, 95% CI 7.40–509.51), white blood count (OR 1.35, 95% CI 1.20–1.52), red cell distribution width-standard deviation (OR 1.22, 95% CI 1.08–1.38), and glomerular filtration rate (OR 0.96, 95% CI 0.94–0.97) were identified as independent risk factors for mortality in patients with diquat. Subsequently, a nomogram with an area under the curve of 0.97 (95% CI: 0.93–1) was developed. Internal bootstrap resampling (1000 repetitions) confirmed the model’s adequate discriminatory power, with an area under the curve of 0.97. Decision curve analysis demonstrated greater net gains for the nomogram, while the clinical impact curves indicated greater predictive validity.

**Conclusion:**

The nomogram model developed in this study using available clinical data enhances the prediction of risk for DQ patients and has the potential to provide valuable clinical insights to guide patient treatment decisions.

**Supplementary Information:**

The online version contains supplementary material available at 10.1186/s12995-024-00416-7.

## Introduction

Acute poisoning is a major public health problem, drugs abuse being the most common, followed by pesticides, while suicide attempts and suicides are the main reasons [1]. At present, all formulations of paraquat have been entirely discontinued in China [2], leading to a gradual increase in the incidence of poisoning involving diquat (DQ) as a replacement [3]. Like for paraquat, there is no specific antidote for DQ. Despite the development of a consensus on the treatment of DQ poisoning in recent years [4], the mortality rate remains high [5]. Furthermore, DQ poisoning can lead to long-term kidney damage in patients [6], and similar cases were found during clinical follow-up, negatively impacting patients’ quality of life.

Theoretically, the concentration of DQ in the blood is considered to be the most reliable indicator of the severity of a patient’s condition, but it is not feasible to perform promptly in primary care facilities. Consequently, by leveraging objective clinical data, it is possible to develop an early, precise, and reliable prognostic assessment system to aid clinicians in ascertaining the extent of poisoning and forecasting patient prognosis, thereby selecting the optimal treatment strategies. Previous studies have investigated the prognostic factors for acute diquat poisoning (ADP) patients, mostly focusing on toxicant intake dose and blood laboratory indices, but have yielded inconsistent results [5, 7–10]. Furthermore, a study has developed a nomogram to determine the risk of death in DQ patients based on vital signs and laboratory results [10]. However, external validation has not been conducted. An optimal prognostic model should offer precise and clinically significant predictions while minimizing the number of variables involved [11].

This study aimed to ascertain the risk factors linked to mortality based on clinic-available variables, including diquat intake dosage, laboratory markers, and clinical characteristics, and construct a nomogram for prognostic prediction in patients. The aim of this study is to accurately assess the risk of death in patients with acute diquat poisoning so that emergency physicians can quickly determine the severity of the patient’s condition and better select treatment strategies.

## Materials and methods

### Study population

The study protocol was conducted with the approval of the West China Fourth Hospital Ethics Committee, Sichuan University [No. HXSY-EC-2,022,116]. Informed consent was not required as no personal data was revealed.

**Fig. 1:**
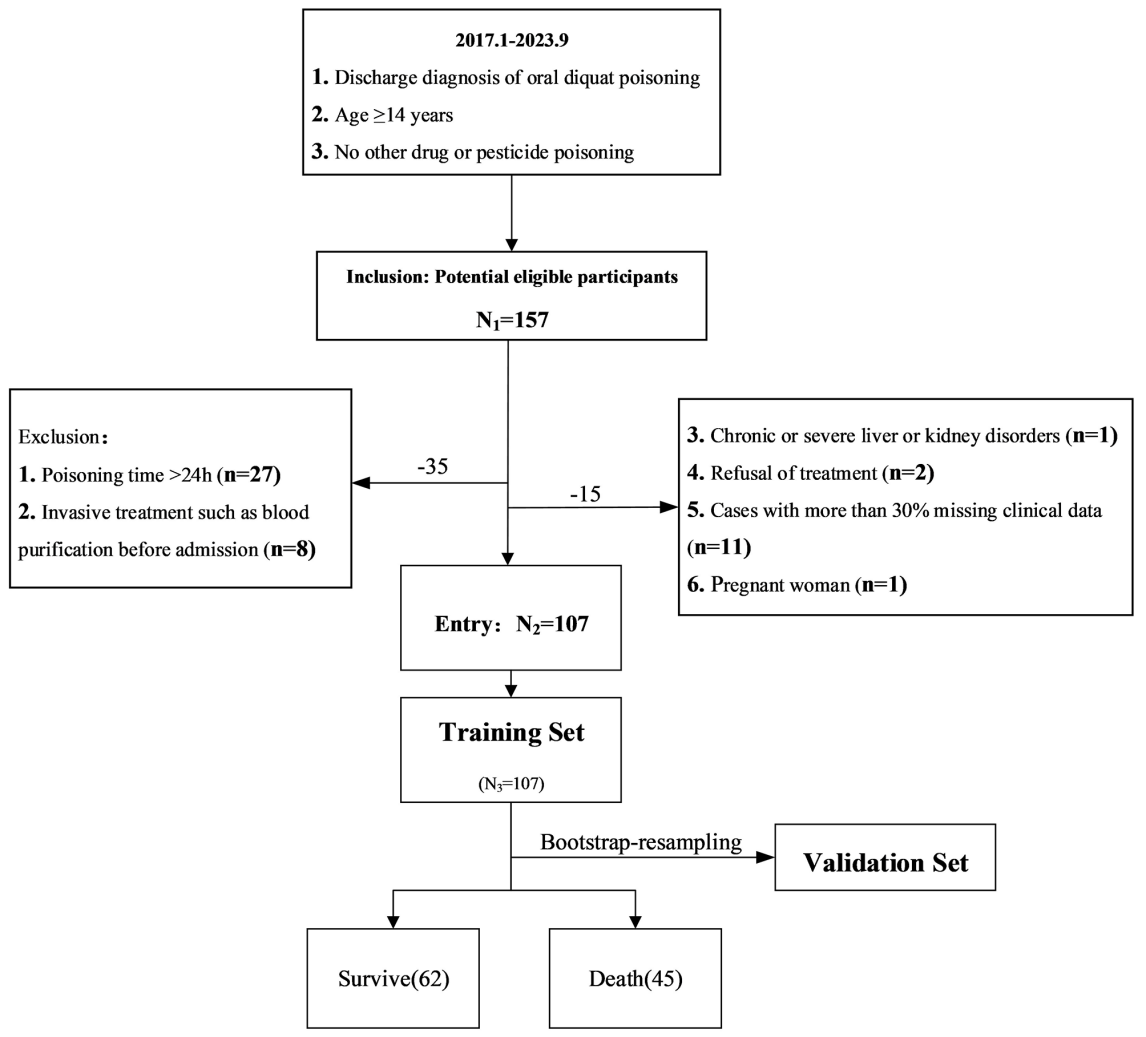
Flow chart for patient selection

Patients who suffered from DQ poisoning and received treatment at a tertiary hospital in Sichuan Province between January 1, 2017, and September 30, 2023, were included in the study. The patients’ prognoses were monitored for 30 days through telephone follow-ups or by reviewing their medical records. Participants were required to meet the following criteria to be included in the study: (1) a discharge diagnosis of oral diquat poisoning, (2) a minimum age of 14 years, and (3) the absence of any other drug or pesticide poisoning. Conversely, individuals were excluded from the study if they met the following criteria: (1) poisoning occurred more than 24 h prior; (2) underwent invasive treatments such as blood purification before admission; (3) suffered from chronic or severe liver or kidney disorders; (4) declined treatment; (5) had more than 30% of clinical data missing; and (6) Pregnant woman (Fig. 1). DQ poisoning diagnosis is based on the patient’s clear history of contact, clinical symptoms, and urine DQ concentration or plasma DQ concentration.

Treatment includes discontinuing DQ absorption, expediting DQ excretion, and providing symptomatic therapy. Initially, gastric lavage with physiological saline was performed, followed by gastrointestinal decontamination using montmorillonite powder and activated charcoal. Next, DQ was eliminated more quickly through increased diuresis and blood purification. Finally, corticosteroids were used for symptomatic treatment to scavenge inflammatory mediators, and vitamin C was used as an antioxidant to scavenge oxygen-free radicals. A HA330 hemoperfusion device was used, with a flow rate of 100–200 ml/min for blood and filtrate. Hemoperfusion is performed every eight hours on the first day, every twelve hours on the second day, and once a day on the third and fourth days, every time for 2 h. The filtrate flow rates for continuous kidney replacement therapy are 1500 to 2000 ml/h, to be administered once a day for 8 to 20 h.

### Study variables

The data of all patients were collected in the medical records, including (a) demographic parameters such as age and sex; (b) exposure such as estimated DQ intake volume, time from DQ exposure to visiting our hospital, and time of first gastric lavage; (c) symptoms 24 h after admission, including blood pressure (BP), heart rate, respiration, fever, bowel sounds, lung rales, nausea and vomiting, abdominal pain, diarrhea, oliguria, irritability, delirium, convulsions, coma, confusion, dyspnea, oliguria and anuria, muscle pain, etc.; and (d) information on the first clinical auxiliary examination, including blood tests for routine coagulation function, liver and kidney function, blood gas analysis, myocardial enzymes, neutrophil-to-lymphocyte ratio, neutrophil-to-lymphocyte and platelet ratio, and the acute physiology and chronic health evaluation score (APACHE II). Some of the indicators exhibited large within-group differences; therefore, the neutrophil-to-lymphocyte and platelet ratios; activated partial thromboplastin time; thrombin time; aspartate aminotransferase; alanine aminotransferase; γ-glutamyl transpeptidase; cholinesterase; creatine kinase; creatine kinase-MB mass; serum creatinine; and lactate dehydrogenase were logarithmically transformed to minimize the effect of outliers and to improve the interpretability of the results of interest. A modified estimated glomerular filtration rate (eGFR) equation based on the Chinese population was used [12].

### Model development and validation

R software was used to address missing data through multiple interpolations, for a total of five interpolations. The interpolation model included all the predictors and outcome variables. After the completion of interpolation, each comprehensive dataset underwent analysis, and the effects were combined using Rubin’s rule to account for the uncertainty introduced by the interpolation method. For each interpolation dataset, variables were selected by applying LASSO regression, and then, independent risk factors were identified using reverse logistic regression (LR). The five interpolated datasets produced five sets of independent risk factor combinations and the final predictor variables were selected from the variables that appeared at least three times in these five combinations. Variables that were incorporated into the LASSO regression model had a significance level of *P* < 0.20 or were deemed clinically significant. To create the final prognostic model, the ultimate predictors were reassessed in each of the five interpolated datasets for regression analyses and then combined with the effect quantity determined by the Rubin rule. Multicollinearity was assessed using the variance inflation factor.

To investigate the model’s robustness and reliability, we used receiver operating characteristic (ROC) curves, accuracy, specificity, sensitivity, and F1 score to assess the model’s differentiation and calibration. We also constructed a decision curve analysis (DCA) curve to determine the model’s clinical applicability and used a nomogram to visualize the risk of death. The 1000 bootstrap resampling method was used to perform internal validation on five datasets. We conducted an analysis of the individuals who received plasma DQ concentrations within this timeframe, assessed its efficacy using the methods described above, and juxtaposed the findings with those of the established model for comparison. We performed an external test of the APACHE II score. Additionally, we conducted sensitivity analyses. First, we evaluated the efficacy of the DQ intake volume by including it in the final model as a continuous and classified variable. Second, we analyzed the odds ratios (ORs) of univariate logistic regression analyses before and after the data were collected. Finally, we compared the nomogram and individual predictors to assess their predictive accuracy.

### Statistical analysis

Qualitative data are presented as frequencies and percentages, while normally distributed quantitative data are presented as the means (standard deviations) or $\stackrel{-}{x}\pm s$, and skewed data are presented as the medians (M_1_∼Q_3_). The normality of the data was primarily assessed using the Shapiro‒Wilk test. Parametric statistical tests, such as t and z tests, were used to analyze continuous data that were normally distributed. Conversely, nonparametric tests, such as the Wilcoxon rank sum test and χ^2^ test, are utilized when normal distribution assumptions are not met, particularly for categorical or ordinal data.

The data were gathered and structured using Excel, while the statistical analysis was conducted using SPSS 27.0 and R 4.3.2 software. R software utilizes various packages, such as glmnet, rms, rmda, scitb, regplot, pROC, reportROC, and mice package.

This article follows the TRIPOD specification [13].

## Results

### General patient information

A total of 107 patients were eventually enrolled in the study, with 62 survivors and 45 deaths after 30 days of poisoning (Fig. 1). The patients had a mean age of 30.97 years, and 63 (58.90%) of them were female. The mean ingested dose was 78.50 ml, with a maximum dose of 500 ml. The mean time from poisoning to admission at our hospital was 7.50 h, and the duration of the first gastric lavage after exposure was 3.38 h. The mean APACHE II score was 12.90. Refer to Table 1 (supplementary material: Table 1) for further details.

### Predictor variables

**Fig. 2:**
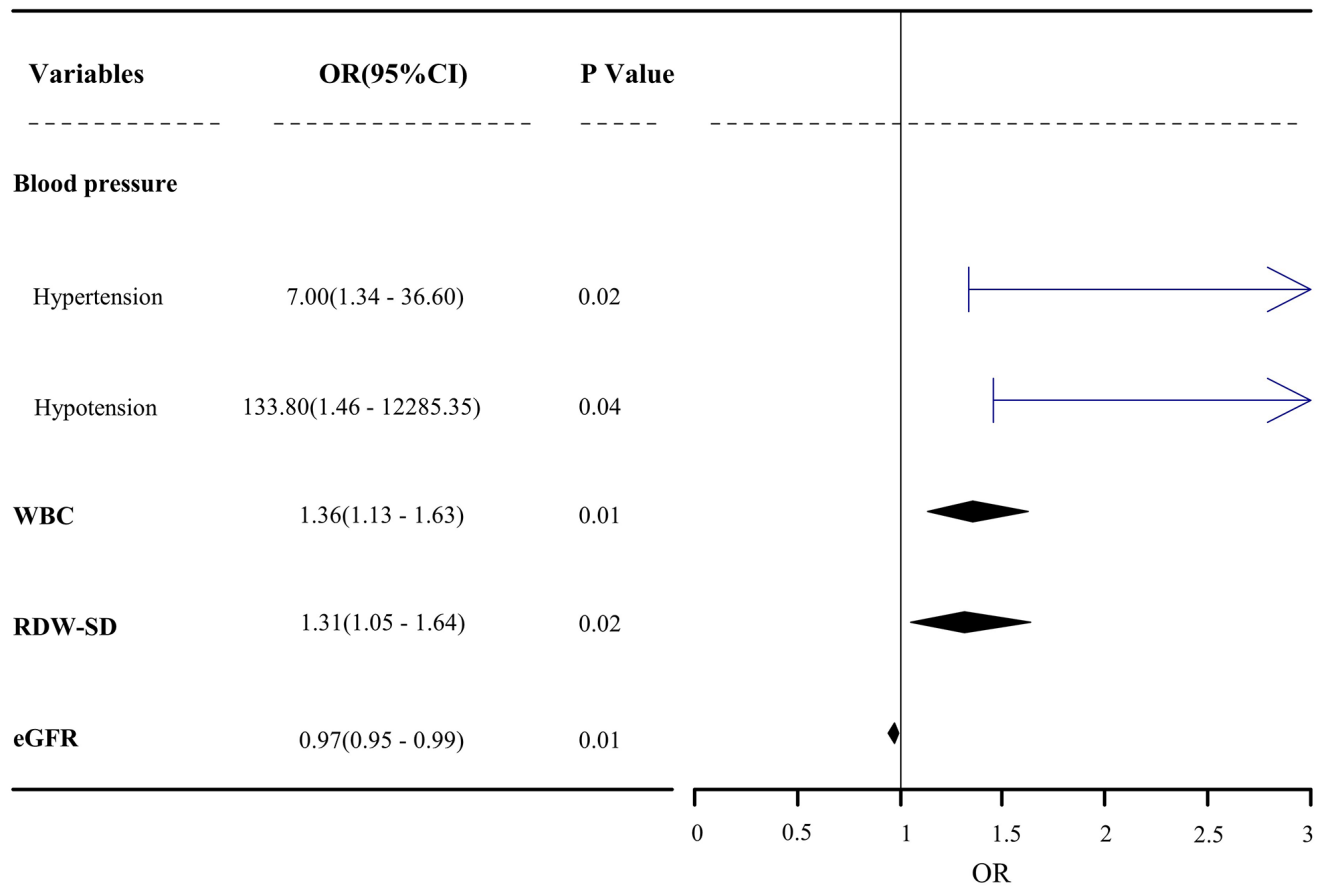
Estimated odds ratios determined in a logistic regression model. Abbreviations: OR: odds ratio; CI: confidence interval; WBC, white blood cell count; RDW-SD, red cell distribution width-standard deviation; eGFR, estimated glomerular filtration rate

A total of 5 variables are missing for more than 30% (supplementary material: Table 2), so we delete variables such as Lac, PH, PaCO_2_, PaO_2_, and SaO_2_. According to the univariate analysis, 53 candidate variables were obtained (*P* < 0.20, except APACHE II). LASSO regression was used to select the feature variables for each dataset, and backward stepwise logistic regression was used to include the feature variables in the multifactor analysis. If a variable was present in the results of three or more interpolation datasets, we retained it. The final model included four predictors: high and low BP at 24 h after admission, first White Blood Cell Count (WBC) on admission, Red Cell Distribution Width - Standard Deviation (RDW-SD), and the eGFR (Fig. 2). Effect sizes and 95% CI were combined using Rubin’s rule across five interpolated datasets. The variables did not have any covariance (variance inflation factor < 2), and the Box-Tidwell method demonstrated a linear relationship between continuous independent variables and logit(P) (*P* > 0.05).

### Construction of the nomogram

**Fig. 3:**
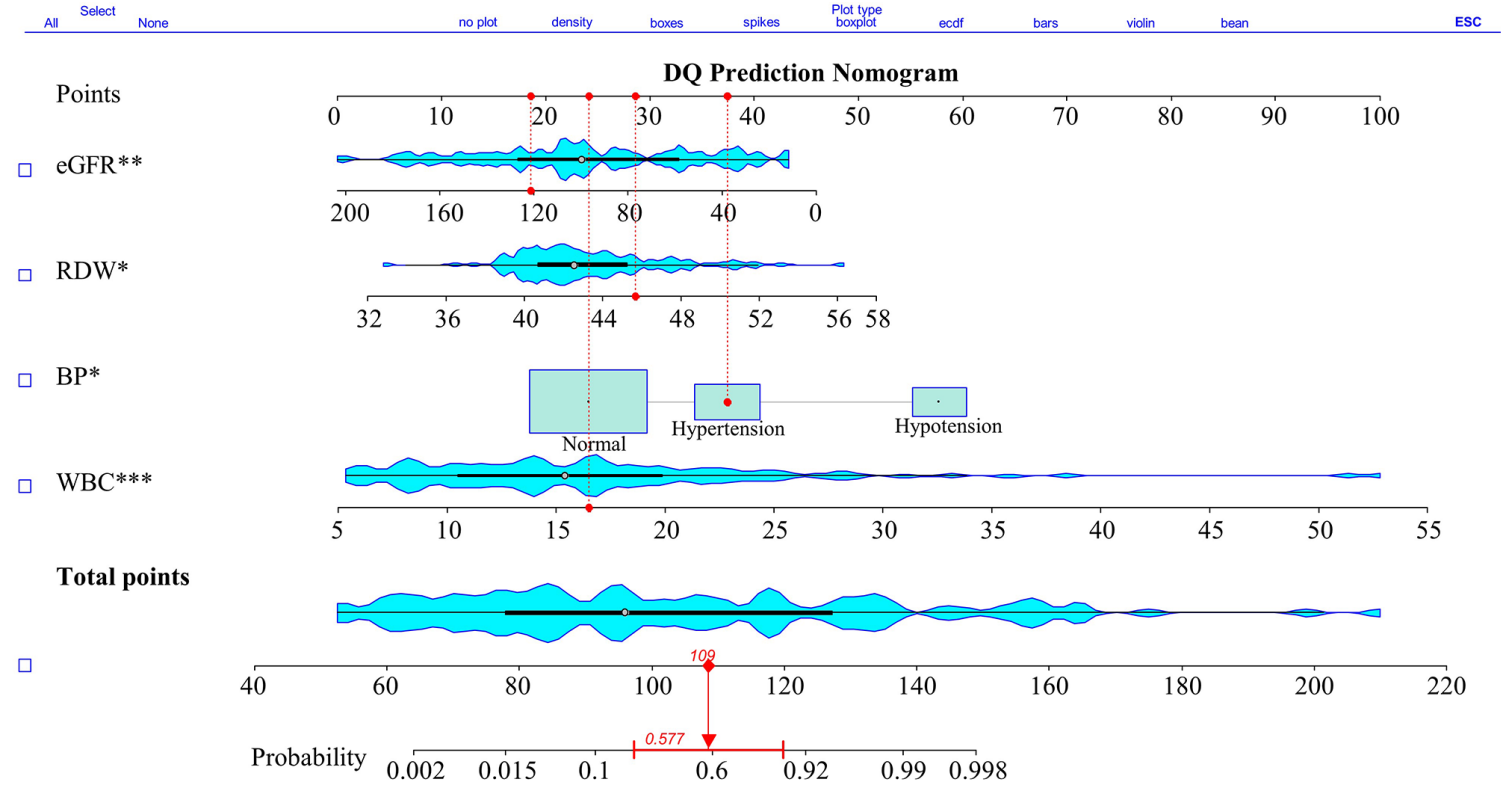
Nomogram for determining the risk of death in acute diquat poisoning patients. The value of each variable was scored on a point scale from 0 to 100, after which the scores for each variable were summed. That sum is located on the total points axis, which enables us to predict the probability of death risk. WBC, white blood cell count; RDW-SD, red cell distribution width-standard deviation; eGFR, estimated glomerular filtration rate; BP, blood pressure; DQ, diquat

Correspondingly, a nomogram was constructed to predict the risk of death in patients with DQ based on the above indices. A higher total score indicates a greater risk of death. A patient who underwent DQ and had a blood test and a WBC of 16.50 × 10^6^/L, an RDW-SD of 45.60, and an eGFR of 121.00 mL/min, as well as an elevated BP that totals a score of 109, had a probability of a poor prognosis of 0.58(0.21, 0.88) (Fig. 3).

### Performance of the nomogram

**Fig. 4:**
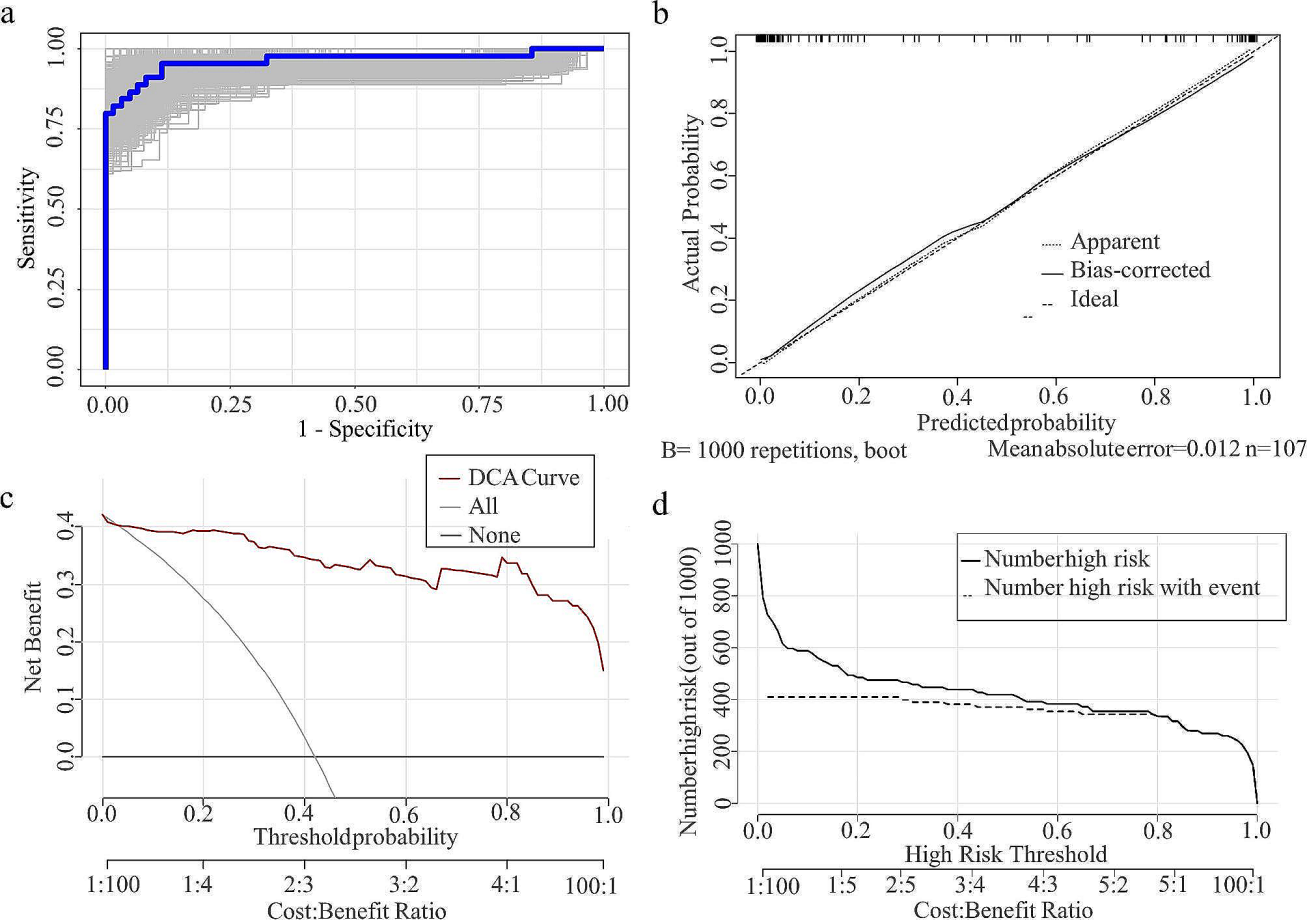
Evaluation of the nomogram model. (**a**) Receiver operating characteristic curves for the nomogram generated using bootstrap resampling (1000 times). (**b**) Nomogram calibration plot. (**c**) Decision curve analysis (DCA) for the prediction model. (**d**) Clinical impact curve for the prediction model

**Fig. 5:**
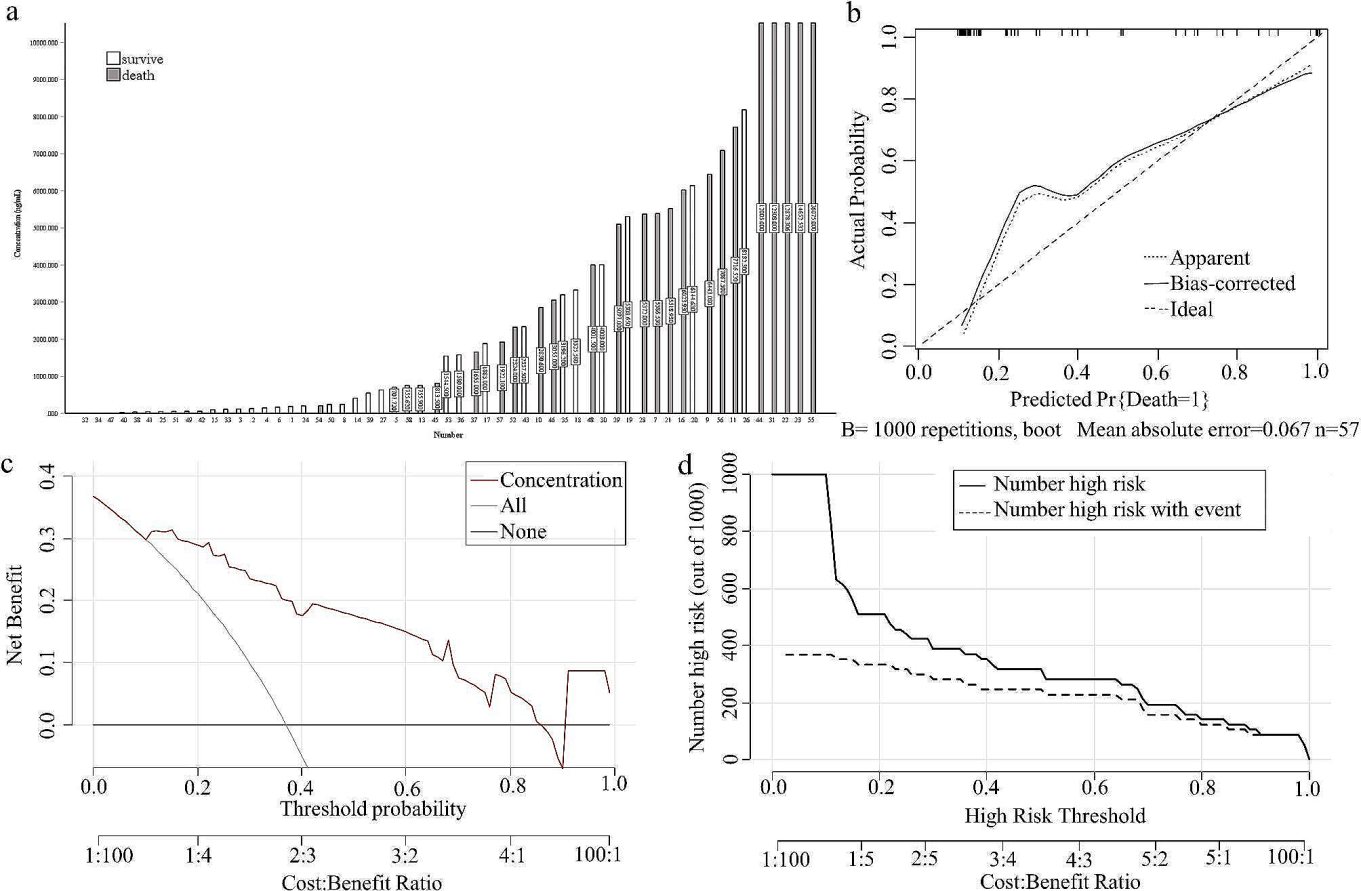
Plasma diquat concentration performance. (**a**) Based on prognosis, relationship between plasma diquat concentration and prognosis; (**b**) Calibration curve for plasma diquat concentration; (**c**) Decision curve analysis for plasma diquat concentration; (**d**) Clinical impact curve for plasma diquat concentration

At the optimal cutoff value of 0.25, the model demonstrated high discrimination, with an area under the curve (AUC) of 0.97 (95% CI = 0.93–1). The accuracy, sensitivity, and specificity were 0.93, 0.98, and 0.89, respectively, with an F1 score of 0.92. To verify the precision of the nomogram, a bootstrap resampling method with 1,000 replications was employed, resulting in an AUC of 0.97 (Fig. 4.a) and an accuracy, sensitivity, and specificity of 0.88, 0.89, and 0.86, respectively, along with a kappa index of 0.75. The closer the apparent or bias-corrected line is to the ideal line, the better the prediction. The calibration curve results showed that the predicted probabilities matched the actual probabilities to a greater extent (apparent line), and the predicted probabilities after correction had a greater degree of consistency with the actual probabilities (bias-corrected line) (Fig. 4. b), with a better fit (R^2^ = 0.82) and a Brier score of only 0.06. The DCA curves display the net gain in comparison to the extremes of both intervention (all) and no intervention (none) for all patients, as well as the predictive model. The higher the net gain is, the more valuable the model. The model is positioned above the None and All lines within the threshold probability of 0.04-1.00, indicating that it can offer greater clinical benefits within this range (Fig. 4. c). The clinical impact curve (CIC) was used to assess the clinical applicability of the risk prediction nomogram. The CIC plot indicates that the dashed line deviates from the solid line in the curve range of 0-0.20. However, when the threshold probability value exceeds 0.20, particularly when it surpasses 0.52, the model accurately predicts the high-risk population that corresponds to the actual population of deaths (Fig. 4.d). This indicates that the model is effective and has a high prediction efficiency. After hospitalization, the plasma DQ concentration was initially found to be significantly lower in the survival group than in the death group and was used to predict patient risk of death, with an AUC of 0.89 (0.81–0.98), an accuracy of 0.83, and a Youden index of only 0.68 (cutoff of 1617.50 ng/mL, sensitivity of 0.91, specificity of 0.78). The calibration curve demonstrated a greater degree of correspondence between the predicted and actual probabilities, resulting in a more accurate prediction but a deviation from the ideal curve (Brier = 0.14, Emax = 0.21) (Fig. 5. b). The DCA curve illustrated that plasma DQ concentrations within the 0.11–0.85 threshold probability had good clinical utility (Fig. 5. c), and the CIC chart showed that the high-risk groups predicted in the model after the risk of 0.68 closely matched those with actual deaths (Fig. 5.d).

### Comparison of nomograms with a single independent predictor

**Fig. 6:**
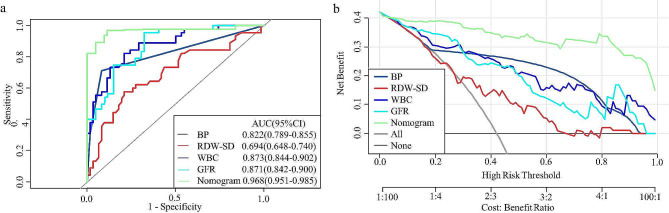
Comparison of Nomograms with a Single Independent Predictor. (**a**) Receiver operating characteristic curves for the nomogram and independent prognostic factors. (**b**) Decision curve analysis of the nomogram and independent predictors. BP, blood pressure; WBC, white blood cell count; RDW-SD, red cell distribution width - standard deviation; eGFR, estimated glomerular filtration rate

The predictive performance of each component comprising the model was assessed individually. The AUCs for BP, WBC, RDW-SD, and the eGFR were 0.82 (95% CI: 0.79–0.86), 0.87 (95% CI: 0.84–0.90), 0.69 (95% CI: 0.65–0.74), and 0.87 (95% CI: 0.84–0.90), respectively. These values were notably lower than the AUCs of the combined model (0.97, 95% CI = 0.95–0.99) (Fig. 6.a). Furthermore, the DCA curves of the combined model also demonstrated superior performance compared to that of the individual independent predictors (Fig. 6. b).

### Sensitivity analysis

#### Univariate logistic regression analyses

Univariate logistic regression analyses were also conducted both before and after the data were collected. The ORs before and after the data were collected were highly consistent, demonstrating the stability of the nomogram (supplementary material: Table 3).

#### Dose as both a continuous and classified variable

**Fig. 7:**
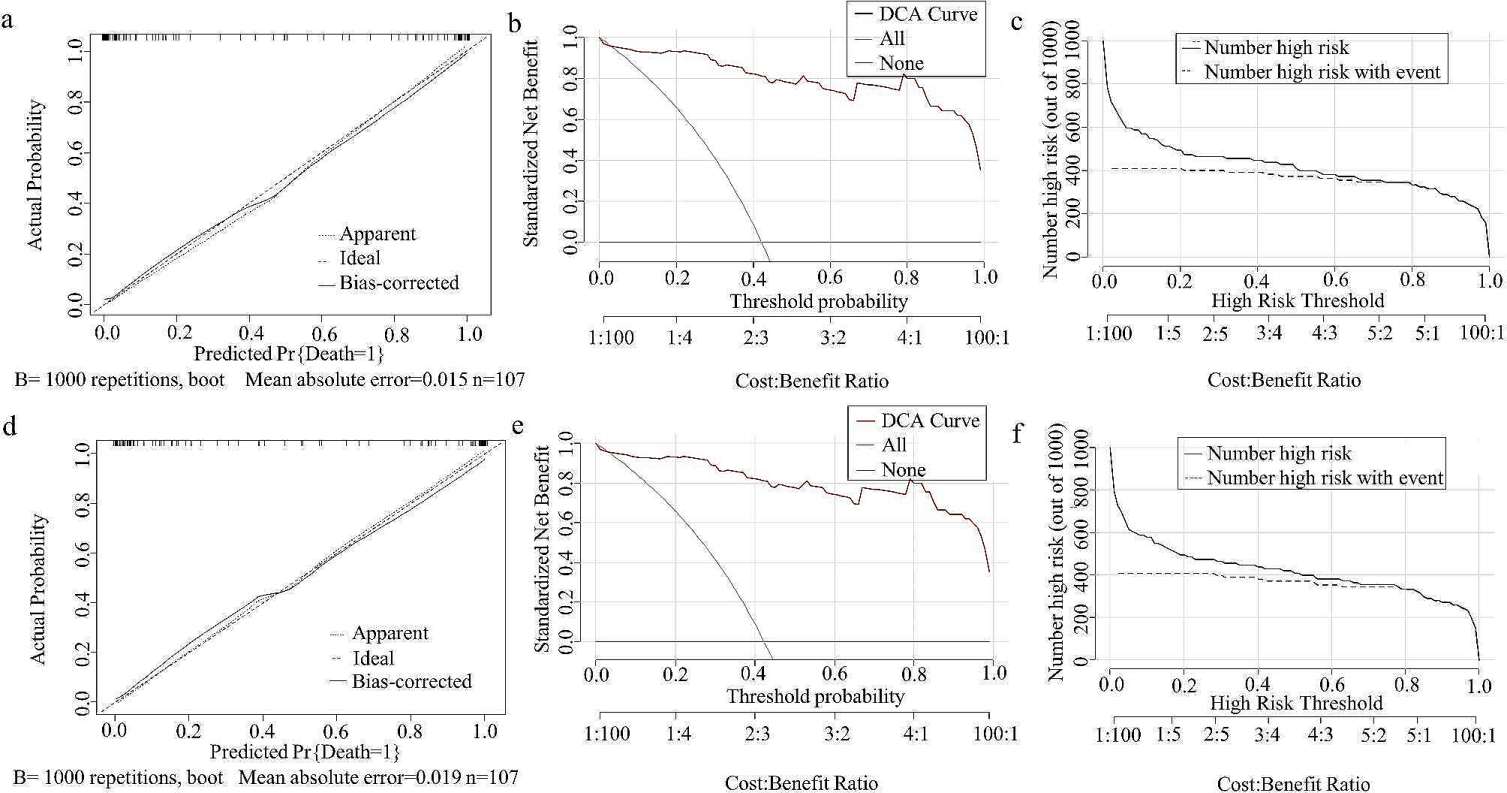
Dose evaluation was performed as a continuous and classified variable. (**a**) Continuous dose calibration plot. (**b**) Decision curve analysis for the continuous dose. (**c**) Clinical impact curve for the continuous dose. (**d**) Classified dose calibration plot. (**e**) Decision curve analysis for the classified dose. (**f**) Clinical impact curve for the classified dose

The DQ intake dosage can serve as a prognostic indicator for patients with DQ. In our study, the AUC of estimated DQ intake was 0.81 (95% CI: 0.77–0.84); the accuracy, sensitivity, and specificity of the assessments were 0.77, 0.80, and 0.74, respectively; and the Youden index was 0.54. Notably, these values are notably inferior to the performance of the other predictive models. Thus, we included the estimated toxic dose as both a continuous and classified variable in the final model. At the optimal cutoff value, the AUC was 0.97 (0.93, 1.00) for both markers, with accuracies of 0.92 and 0.93, sensitivities of 0.96 and 0.98, specificities of 0.89, and F1 scores of 0.91 and 0.92. However, the calibration curve (brier = 0.06), DCA (threshold = 0.04-1.00), and CIC did not significantly improve the performance (Fig. 7). Considering the number of variables, the ingested toxic dose was ultimately not included in the model.

## Discussion

There is no known antidote for diquat poisoning, and high doses often cause irreversible effects [14]. The incidence of diquat poisoning has increased over the years, with suicide being the most common cause of poisoning [15]. Mortality rates have been reported to vary from 43.00% [5] to 60.00% [15], while the mortality rate was 42.05% in the present study. In this research, a comprehensive array of latent influencing factors derived from expert experience and findings from prior research were gathered to evaluate their association with the risk of mortality. Nomogram models can be used to visualize influencing factors clearly and effectively. In this study, we analyzed early clinical data from 107 patients diagnosed with diquat poisoning. Using stepwise multifactorial logistic regression analyses, we identified four prognostic factors: BP, WBC, RDW-SD, and eGFR. Then we developed a nomogram that predicted the risk of death in patients after 30 days of poisoning. The generalizability of the nomogram relies on patients receiving identical treatment. A lower eGFR, higher WBC and RDW-SD, and abnormal BP are correlated with a heightened risk of death. The AUC, confusion matrix, calibration curve, Brier score, and DCA confirmed that the model has good discriminative ability and excellent calibration ability, and internal verification was carried out by bootstrap resampling.

We also used the data to externally validate the APACHE II scoring model [8], which achieved an AUC of up to 0.95 (95% CI = 0.90–0.99), an accuracy of 0.90, a sensitivity of 0.87, a specificity of 0.92, a Youden index of 0.79, and an F1 score of 0.88.

The study results indicate that the deceased patients had significantly greater WBC and RDW-SD counts than did the surviving patients (*P* < 0.01). Additionally, the eGFR was significantly lower in the deceased group (*P* < 0.01), and there was a significantly greater number of patients with abnormal BP. A systemic inflammatory response may occur in patients with DQ [16]. Previous studies have also reported a close association between early elevation of WBC and adverse prognosis, suggesting that WBC may serve as a prognostic factor, as confirmed by the present study [5, 10, 17]. The RDW has recently been found to have a strong predictive capacity for the risk of death and adverse outcomes of other infectious and serious diseases, such as COVID-19 [18], acute respiratory obstruction syndrome [19], and sepsis [20]. Studies of ICU patients have shown that the RDW is an independent risk factor for death and is significantly and independently related to mortality [21]. A study also revealed that an increased RDW can serve as an independent predictor of 30-day mortality in patients with organophosphorus poisoning [22]. An increase in RDW may be attributed to various metabolic abnormalities, such as oxidative stress, inflammation, poor nutritional status, and high blood pressure [21]. DQ poisoning can cause oxidative stress-related damage to the body, activate the NF-κB pathway, and induce an inflammatory response in the body, affecting red blood cell stability and survival [23]. Furthermore, there is a strong, graded, and independent correlation between RDW and eGFR [21]. Clinically, ADP patients are characterized by multiorgan damage, primarily involving the kidneys and central nervous system [17], and some patients have complications such as anemia [24], systemic inflammatory reactions [16], and acute respiratory distress syndrome [5] in the later stages. Glomerular filtration rate is the rate at which plasma is filtered to produce ultrafiltrate. Therefore, measurement of the simpler eGFR is widely used in the clinical front line, as is its ability to reflect the magnitude and direction of the true GFR serving during acute kidney injury [25]. DQ in the bloodstream is mostly excreted by the kidney [26], so the occurrence of kidney damage is closely related to a decrease in the ability to remove toxins, which may have a significant impact on patient prognosis. Kidney injury is a prominent and early effect of ADP, with an incidence rate as high as 82.95% [27], and is characterized mainly by a significant decrease in the eGFR and delayed recovery of renal function [6]. A biopsy reveals acute tubular necrosis in a patient with diquat poisoning[6]. However, tubular recovery after acute kidney injury is vital for recovery of kidney function, including improvement of GFR, and likely determines which patients fully recover from acute kidney injury or progress to chronic kidney disease [28], which may also explain why some patients have a longer duration of kidney injury. Many studies have confirmed that DQ can cause nephrotoxicity [29, 30], and its early onset may indicate a poor prognosis [31]. Alterations in an individual’s blood pressure can serve as an indicator of the individual’s fundamental physiological state. Hypertensive patients experience accelerated blood flow, which can lead to faster toxin distribution to organs. The blood pressure of patients who died due to diquat poisoning decreased within 8 to 30 h after ingestion, and death occurred within 2 to 14 h after the drop [5]. Patients with secondary acute hypotension may develop organ hypoperfusion [32] and cannot promptly supply enough nutrition and oxygen to vital organs in the body, which worsens the patient’s condition and creates a vicious cycle.

The concentration of toxicants in the blood is considered to be the gold standard for theoretically evaluating a patient’s condition [33]. We recorded the plasma DQ concentrations at the time of admission in 57 patients because some patients were tested only for urine DQ concentrations. The risk of death is positively correlated with the concentration of toxins in the body, which increases as the concentration increases (Fig. 5. a). The median plasma DQ concentration was 221.40[56.00,1571.12] in the surviving patients (*n* = 36) and 5386.53[2587.30,9860.76] in the death group (*n* = 21), which was significantly higher than that of the surviving group (*P* < 0.05). Compared to the model described in the text, the plasma DQ concentration used to predict patient prognosis had an AUC of 0.89 (95% CI: 0.81–0.98), which was lower than the AUC of the aforementioned model. This finding was associated with a decrease in accuracy, and the results of the 1000 bootstrap replicates were also not as good (ACC = 0.80, kappa = 0.56). The severity index of diquat poisoning (SIDP) is calculated by multiplying the plasma DQ concentration by the duration of poisoning [8]. Additionally, the SIDP used to predict patient prognosis had an AUC (0.88 [0.78, 0.98]) was less than the single concentration, the ACC (0.84), and the Youden index (0.69) were slightly greater, while the overall result was inferior to the concentration model. To determine the type and severity of poisoning, the blood concentration of the poison is considered the gold standard. A retrospective cohort study of 50 patients confirmed a relationship between plasma DQ concentrations and in-hospital mortality (AUC = 0.97 [0.91, 1.00], cutoff = 3516.89 ng/ml [sensitivity, 90.90%; specificity, 96.00%]) [33]. In contrast, some patients did not receive complete continuous treatment, which may have affected the study’s results. However, it can be challenging to measure toxicant concentrations in patient body fluids accurately in primary health centers and some hospitals due to the lack of access to high-precision equipment.

The study also revealed a distinct correlation between the dose taken and mortality (*P* < 0.01). The group of individuals who died ingested a significantly greater dosage than the group of survivors did, consistent with findings from previous research [10, 17], and some studies have included it as a prognostic influencing factor or even constructed relevant models [10, 34, 35]. However, this variable was not included in the present study after analysis. Considering toxic doses did not improve the predictive effectiveness of the model. Currently, the most precise approach for determining the dosage of orally ingested poisons is the oral water method [36]. The method of operation is to prepare a bottle of mineral water (250 ml) with a diameter similar to that of the pesticide bottle, simulate the situation of taking poison, where the patient takes the same number of mouthfuls of mineral water, and estimate the dose of poison taken by the patient through the measurement of the amount of water remaining in the bottle. Nonetheless, this method may be challenging to use for certain critically ill patients because the information provided by family members may lack accuracy, and patients may also experience recall bias, leading to a more subjective assessment. Therefore, considering the number of variables, we still chose the final model without the toxic dose.

In this study, a nomogram prediction model of mortality risk in patients with acute diquat poisoning was established, combining objective indicators and patients’ status and assigning a score to each risk factor to provide the corresponding probability of mortality risk, which can rapidly identify patients with critical diquat poisoning at an early stage and assess the risk of mortality, help clinicians choose the most optimal treatment decision. If the model predicted a low risk of death for the patient, giving the current standardized treatment would improve the prognosis. Conversely, even with standardized treatment, those who are at high risk of mortality may not always improve their prognosis. Thus, economic costs and predicted values should be appropriately taken into account, and suitable steps should be taken to lessen their suffering. However, the model’s generalization and extrapolation accuracy remain to be verified since the study was not externally validated. Additionally, the study has some limitations. First, the small sample size and single-center retrospective design of this study limit the amount of data collected, which may introduce bias that affects the universality of the research results. Furthermore, despite the inclusion of many variables in this study to cover all the influencing factors as much as possible, certain specificity indicators identified in prior research, such as body mass index [37], neutrophil gelatinase-associated lipocalin [38, 39], serum toxicant concentration [33], and lactate concentration [10], were not adequately captured for various reasons. Finally, although the prediction model demonstrated a certain degree of accuracy, the initial clinical data within 24 h after admission did not fully reflect the degree of organ damage caused by DQ. Moreover, certain patients did not manifest abnormal clinical symptoms or atypical blood test results upon admission [14]. Therefore, it is necessary to monitor and observe specific indicators during follow-up, and the construction of the final prediction model requires further exploration and analysis.

## Conclusion

In conclusion, through the integration of clinical characteristics and standard blood test findings, we developed a nomogram utilizing 24-hour BP, WBC, RDW-SD, and eGFR parameters that was simpler and earlier than the APACHE II score. Patients with diquat poisoning in the early stages do not exhibit specific clinical features, and routine blood test results also reveal varying manifestations. Thus, early prediction of the prognosis of ADP patients by the nomogram can help clinicians assess the severity of the disease and adjust clinical treatment while minimizing unnecessary use of medical resources, improving the survival rates of patients, and reducing the overall impact of the disease.

### Electronic supplementary material

Below is the link to the electronic supplementary material.

Supplementary Material 1

## Data Availability

All the data generated or analyzed during this study are included in this published article, and the other data and materials were obtained from the Department of Emergency Medicine, West China Fourth Hospital. The data analyzed in this study are subject to the following licenses and limitations: the dataset used in the study is not publicly available but is available from the corresponding author, Yu Du, upon reasonable request.

## Abbreviations

ADP: Acute Diquat Poisoning
APACHE II: Acute Physiology and Chronic Health Evaluation score II
AUC: Area Under the Curve
BP: Blood Pressure
CI: Confidence Interval
CIC: Clinical Impact Curve
COVID-19: Coronavirus Disease 2019
DCA: Decision Curve Analysis
DQ: Diquat
eGFR: Estimated Glomerular Filtration Rate
OR: Odds Ratio
RDW-CV: Red Cell Distribution Width - Coefficient of Variation
RDW-SD: Red Cell Distribution Width - Standard Deviation
ROC: Receiver Operating Characteristic
WBC: White Blood Cell Count
