# Supplementary material for: Construction of a mortality risk prediction model for patients with acute diquat poisoning based on clinically accessible data
Source: J Occup Med Toxicol. 2024 May 21;19:20. doi: 10.1186/s12995-024-00416-7 (PMC11110376; doi:10.1186/s12995-024-00416-7)
Supplement: Supplementary file 1 — Supplementary Material 1 [file 12995_2024_416_MOESM1_ESM.docx]

**Supplement**

Table.1 **Baseline characteristics**

Table.2 **Missing value analysis**

Table.3 **ORs of univariate logistic regression analysis before and after filling in the data**

Table.4 **Variable definition table and Variable unit**

**Table 1: Baseline characteristics**

| **Baseline** | **Total** | **Survive** | **Death** | **P** |
| --- | --- | --- | --- | --- |
| Number | 107 | 62 | 45 |  |
| Demographic parameters(n=107) |  |  |  |  |
| Age (years, median [IQR]) | 27.00[19.00-39.00] | 25.00 (19.00-32.00) | 30.00 (20.00-49.00) | 0.020 |
| Gender (%) |  |  |  |  |
| male | 44(41.12) | 19 (30.65) | 25 (55.56) | 0.010 |
| female | 63(58.90) | 43 (69.35) | 20 (44.44) |  |
| Exposure(n=107) |  |  |  |  |
| Dose (mL, [IQR]) | 50.00[20.00-100.00] | 30.00 (20.00-57.50) | 100.00 (70.00-150.00) | <0.001 |
| Time to the hospital (h, [IQR]) | 5.00[3.00-10.00] | 6.00 (3.00-10.00) | 4.00 (3.00-9.00) | 0.18 |
| Time of first gastric lavage (h, [IQR]) | 2.00[1.00-3.00] | 2.00 (1.00-4.00) | 2.00 (1.00-3.00) | 0.12 |
| Symptoms 24 hours after admission(n=107) |  |  |  |  |
| Blood pressure (%) |  |  |  | <0.001 |
| Normal | 70(65.40) | 57(91.94) | 13(28.89) |  |
| Hypertension | 22(20.60) | 4(6.45) | 18(40.00) |  |
| Hypotension | 15(14.00) | 1(1.61) | 14(31.11) |  |
| Respiration (%) |  |  |  | 0.002 |
| Normal | 20(18.70) | 18 (29.03) | 2 (4.44) |  |
| Tachypnea | 61(57.00) | 28 (45.16) | 33 (73.33) |  |
| Slowing | 6(5.60) | 5 (8.06) | 1 (2.22) |  |
| Slow down after a hurry | 20(18.70) | 11 (17.74) | 9 (20.00) |  |
| Heart rate (%) |  |  |  | <0.001 |
| Normal | 57(53.30) | 50 (80.65) | 7 (15.56) |  |
| High heart rate | 34(31.80) | 10 (16.13) | 24 (53.33) |  |
| Low heart rate | 3(2.80) | 1 (1.61) | 2 (4.44) |  |
| High first and then low | 13(12.10) | 1 (1.61) | 12 (26.67) |  |
| Fever (%) |  |  |  | 1.0 |
| No | 99(92.50) | 57 (91.94) | 42 (93.33) |  |
| Yes | 8(7.50) | 5 (8.06) | 3 (6.67) |  |
| Bowel sounds (%) |  |  |  | 0.007 |
| Normal | 81(75.70) | 51 (82.26) | 30 (66.67) |  |
| Weakening | 6(5.60) | 0 (0.00) | 6 (13.33) |  |
| Active | 20(18.70) | 11 (17.74) | 9 (20.00) |  |
| Lung rales (%) |  |  |  | <0.001 |
| No | 95(88.80) | 61 (98.39) | 34 (75.56) |  |
| Yes | 12(11.20) | 1 (1.61) | 11 (24.44) |  |
| Vomiting (%) |  |  |  | 0.007 |
| No | 23(21.50) | 19 (30.65) | 4 (8.89) |  |
| Yes | 84(78.50) | 43 (69.35) | 41 (91.11) |  |
| Abdominal pain (%) |  |  |  | 0.18 |
| No | 65(60.75) | 41 (66.13) | 24 (53.33) |  |
| Yes | 42(39.30) | 21 (33.87) | 21 (46.67) |  |
| Diarrhea (%) |  |  |  | 0.53 |
| No | 84(78.50) | 50 (80.65) | 34 (75.56) |  |
| Yes | 23(21.50) | 12 (19.35) | 11 (24.44) |  |
| Oliguria and Anuria (%) |  |  |  | <0.001 |
| No | 78(72.90) | 53 (85.48) | 25 (55.56) |  |
| Yes | 29(27.10) | 9 (14.52) | 20 (44.44) |  |
| Dysphoria (%) |  |  |  | <0.001 |
| No | 81(75.70) | 57 (91.94) | 24 (53.33) |  |
| Yes | 26(24.30) | 5 (8.06) | 21 (46.67) |  |
| Delirium (%) |  |  |  | 0.009 |
| No | 98(91.59) | 61 (98.39) | 37 (82.22) |  |
| Yes | 9(8.40) | 1 (1.61) | 8 (17.78) |  |
| Convulsions (%) |  |  |  | <0.001 |
| No | 98(91.59) | 62 (100.00) | 36 (80.00) |  |
| Yes | 9(8.40) | 0 (0.00) | 9 (20.00) |  |
| Comatose (%) |  |  |  | <0.001 |
| No | 94(87.85) | 62 (100.00) | 32 (71.11) |  |
| Yes | 13(12.10) | 0 (0.00) | 13 (28.89) |  |
| Mental confusion (%) |  |  |  | <0.001 |
| No | 88(82.24) | 61 (98.39) | 27 (60.00) |  |
| Yes | 19(17.80) | 1 (1.61) | 18 (40.00) |  |
| Dyspnea (%) |  |  |  | <0.001 |
| No | 91(85.05) | 61 (98.39) | 30 (66.67) |  |
| Yes | 16(15.00) | 1 (1.61) | 15 (33.33) |  |
| Muscular pain (%) |  |  |  | 0.27 |
| No | 98(91.59) | 59 (95.16) | 39 (86.67) |  |
| Yes | 9(8.40) | 3 (4.84) | 6 (13.33) |  |
| Laboratory tests |  |  |  |  |
| White blood cell count | 15.36[10.36-19.92] (n=107) | 12.33 (8.34-15.18) | 20.61 (16.67-25.40) | <0.001 |
| Neutrophil | 13.63[8.30-18.12] (n=107) | 10.22 (6.69-14.31) | 18.49 (14.38-24.23) | <0.001 |
| Lymphocyte | 1.04[0.73-1.58] (n=107) | 1.02 (0.76-1.52) | 1.25 (0.57-1.70) | 0.92 |
| Monocyte | 0.48[0.35-0.76] (n=107) | 0.42 (0.29-0.56) | 0.70 (0.47-0.85) | <0.001 |
| NEUT (%) | 89.5[82.60-92.80] (n=107) | 87.80 (76.62-91.07) | 91.40 (86.00-94.30) | 0.004 |
| LYM (%) | 7.5[40-13.20] (n=107) | 9.50 (5.10-15.98) | 4.00 (2.80-9.90) | <0.001 |
| MONO (%) | 3.8[2.30-5.10] (n=107) | 3.50 (2.62-4.95) | 4.20 (2.20-5.40) | 0.95 |
| Red blood cell count | 4.64[4.40-5.37] (n=107) | 4.52 (4.31-4.83) | 5.30 (4.61-5.59) | <0.001 |
| Hemoglobin | 144.84±19.62(n=107) | 136.61±15.55 | 156.18±19.10 | <0.001 |
| Hematocrit | 42.90[39.50-48.30] (n=107) | 40.35 (38.38-43.53) | 47.00 (43.10-51.50) | <0.001 |
| Red Cell Distribution Width - Coefficient of Variation (%) | 13.10[12.50-13.70] (n=107) | 13.00 (12.50-13.40) | 13.50 (12.70-14.00) | 0.041 |
| Red cell distribution width - standard deviation | 42.50[40.70-45.20] (n=107) | 41.80 (40.50-43.53) | 44.20 (41.70-47.30) | <0.001 |
| Platelet count | 230.00[190.00-290.00] (n=107) | 231.00 (195.50-290.75) | 218.00 (186.00-269.00) | 0.50 |
| Neutrophil to lymphocyte and platelet ratio | 4.59[2.44-,9.64] (n=107) | 4.14 (2.19-7.22) | 7.69 (3.87-14.36) | <0.001 |
| Mean platelet volume | 10.80[9.70-12.00] (n=107) | 10.90 (9.70-11.65) | 10.70 (9.90-12.30) | 0.46 |
| Plateletocrit | 0.25[0.21-0.30] (n=107) | 0.25 (0.21-0.30) | 0.25 (0.21-0.30) | 0.87 |
| Platelet Large Cell Count | 76.00[58.00-86.00] (n=107) | 74.50 (56.50-84.75) | 78.00 (65.00-94.00) | 0.25 |
| Platelet Large Cell Ratio | 33.12±12.11(n=107) | 31.89±10.25 | 34.82±14.23 | 0.24 |
| Neutrophil to lymphocyte ratio | 11.68[5.40-23.07] (n=107) | 9.19 (4.54-18.01) | 20.23 (8.54-32.74) | <0.001 |
| Monocyte-to-Lymphocyte Ratio | 0.43[0.27-0.82] (n=107) | 0.35 (0.25-0.60) | 0.74 (0.32-1.17) | <0.001 |
| Prothrombin time | 11.55[10.8-12.53] (n=106) | 12.20 (11.20-12.90) | 11.00 (10.40-11.70) | <0.001 |
| International normalized ratio | 1.04[0.96-1.13] (n=106) | 1.08 (1.02-1.17) | 0.98 (0.93-1.05) | <0.001 |
| Activated partial thromboplastin time | 29.25[25.35-33.35] (n=106) | 29.60 (25.80-33.30) | 28.50 (24.10-32.20) | 0.26 |
| Thrombin time | 15.60[13.78-16.93] (n=106) | 15.20 (13.60-17.00) | 15.90 (14.10-16.80) | 0.65 |
| Fibrinogen | 2.74±0.75(n=106) | 2.61±0.67 | 2.91±0.83 | 0.046 |
| Total Protein | 79.54±9.68(n=106) | 78.48±8.36 | 81.04±11.21 | 0.18 |
| Albumin | 47.15[43.78-53.82] (n=106) | 46.65 (44.05-48.68) | 48.15 (43.77-51.78) | 0.19 |
| Globulin | 31.7[28.48-36.10] (n=106) | 31.00 (27.72-33.60) | 33.15 (29.93-38.88) | 0.029 |
| Albumin-globulin ratio | 1.49±0.27(n=106) | 1.52±0.26 | 1.45±0.28 | 0.18 |
| 1/Albumin-globulin ratio | 0.67[0.59-0.77] (n=106) | 0.67 (0.59-0.76) | 0.68 (0.62-0.83) | 0.20 |
| Alanine aminotransferase | 24.50[16.75-41.75] (n=106) | 20.00 (14.00-27.75) | 40.00 (23.75-118.00) | <0.001 |
| Aspartate aminotransferase | 31.50[23.00-53.00] (n=106) | 27.00 (21.00-32.00) | 55.50 (37.00-155.50) | <0.001 |
| Total bilirubin | 15.95[10.25-23.00] (n=106) | 14.95 (9.20-20.75) | 18.95 (12.07-24.20) | 0.045 |
| Direct bilirubin | 0.00[0.00,6.05] (n=106) | 0.00 (0.00-6.00) | 1.50 (0.00-6.40) | 0.77 |
| Indirect Bilirubin | 12.00[7.68,16.15] (n=106) | 11.05 (7.00-15.25) | 13.50 (9.72-17.40) | 0.080 |
| γ-glutamyl transpeptidase | 20.00[14.00-32.00] (n=103) | 16.00 (12.00-22.50) | 26.00 (18.50-47.00) | <0.001 |
| Alkaline phosphatase | 84.00[60.00-102.00] (n=103) | 80.50 (58.75-89.25) | 86.00 (67.50-107.50) | 0.047 |
| Carbon dioxide combining power | 19.79±5.41(n=85) | 21.99±4.56 | 16.63±4.99 | <0.001 |
| Estimated Glomerular filtration rate | 97.88±46.47(n=104) | 121.97±38.54 | 63.70±33.69 | <0.001 |
| Blood urea nitrogen | 5.38[3.91-7.09] (n=106) | 4.85 (3.61-6.17) | 6.75 (4.87-8.41) | <0.001 |
| Serum creatinine | 75.25[56.28,120.80] (n=106) | 63.00 (51.60-79.00) | 120.40 (81.00-208.00) | <0.001 |
| Cholinesterase | 7896.50[6573.00-9332.25] (n=78) | 7440.00 (6336.25-8233.00) | 8986.50 (7393.25-10414.50) | <0.001 |
| Creatine kinase | 112.00[73.00-290.00] (n=85) | 94.00 (67.00-162.00) | 220.00 (100.75-658.25) | <0.001 |
| Creatine kinase-MB Mass | 2.22[0.84-5.37] (n=90) | 1.75 (0.72-2.65) | 4.98 (1.17-11.53) | <0.001 |
| Lactate dehydrogenase | 217.00[166.500-308.75] (n=82) | 173.50 (150.00-219.75) | 375.00 (232.25-850.50) | <0.001 |
| Na^+^ | 137.40[136.0-139.80] (n=89) | 137.35 (136.00-139.80) | 138.00 (135.00-139.70) | 0.78 |
| K^+^ | 3.60±0.55(n=89) | 3.64±0.33 | 3.56±0.72 | 0.52 |
| Cl^-^ | 105.00[102.63-107.08] (n=89) | 105.00 (103.00-106.75) | 105.00 (102.00-107.90) | 0.93 |
| Ca^2+^ | 1.19[1.13-2.19] (n=88) | 1.19 (1.15-2.08) | 1.20 (1.12-2.28) | 0.87 |
| APACHE Ⅱ | 7.00[3.00-19.00] (n=107) | 3.00 (2.00-6.00) | 24.00 (14.00-37.00) | <0.001 |

**Table 2: Missing value analysis**

| Variables | n | Missing | |
| --- | --- | --- | --- |
|  |  | count | % |
| Prothrombin time | 106 | 1 | 0.9 |
| International normalized ratio | 106 | 1 | 0.9 |
| Activated partial thromboplastin time | 106 | 1 | 0.9 |
| Thrombin time | 106 | 1 | 0.9 |
| Fibrinogen | 106 | 1 | 0.9 |
| Total bilirubin | 106 | 1 | 0.9 |
| Albumin | 106 | 1 | 0.9 |
| Globulin | 106 | 1 | 0.9 |
| Alanine aminotransferase | 106 | 1 | 0.9 |
| Aspartate aminotransferase | 106 | 1 | 0.9 |
| Total bilirubin | 106 | 1 | 0.9 |
| Direct bilirubin | 106 | 1 | 0.9 |
| Indirect Bilirubin | 106 | 1 | 0.9 |
| γ-glutamyl transpeptidase | 103 | 4 | 3.7 |
| Alkaline phosphatase | 103 | 4 | 3.7 |
| Cholinesterase | 78 | 29 | 27.1 |
| Creatine kinase | 85 | 22 | 20.6 |
| Creatine kinase-MB Mass | 90 | 17 | 15.9 |
| Carbon dioxide combining power | 85 | 22 | 20.6 |
| Blood urea nitrogen | 106 | 1 | 0.9 |
| Serum creatinine | 106 | 1 | 0.9 |
| Estimated Glomerular filtration rate | 104 | 3 | 2.8 |
| Lactate dehydrogenase | 82 | 25 | 23.4 |
| PH | 71 | 36 | 33.6 |
| Partial Pressure of Carbon Dioxide | 71 | 36 | 33.6 |
| Partial Pressure of Oxygen | 70 | 37 | 34.6 |
| Oxygen saturation | 59 | 48 | 44.9 |
| Na^+^ | 89 | 18 | 16.8 |
| K^+^ | 89 | 18 | 16.8 |
| Cl^-^ | 89 | 19 | 17.8 |
| Ca^2+^ | 88 | 19 | 17.8 |
| Lactate | 63 | 44 | 41.1 |

**Table 3: ORs of univariate logistic regression analysis before and after filling in the data**

| **Variables** | **Before** | | **After** | |
| --- | --- | --- | --- | --- |
|  | **OR (95%CI)** | **P** | **OR (95%CI)** | **P** |
| Age | 1.04 (1.01 - 1.07) | 0.007 | 1.04 (1.01 - 1.07) | 0.007 |
| Dose | 1.02 (1.01 - 1.03) | <0.001 | 1.02 (1.01 - 1.03) | <0.001 |
| Time to the hospital | 0.97 (0.91 - 1.03) | 0.34 | 0.97 (0.91 - 1.03) | 0.34 |
| Time of first gastric lavage | 0.87 (0.75 - 1.01) | 0.07 | 0.87 (0.75 - 1.01) | 0.065 |
| White blood cell count | 1.35 (1.20 - 1.52) | <0.001 | 1.35 (1.20 - 1.52) | <0.001 |
| Neutrophil | 1.26 (1.15 - 1.38) | <0.001 | 1.26 (1.15 - 1.38) | <0.001 |
| Lymphocyte | 1.30 (0.80 - 2.13) | 0.29 | 1.30 (0.80 - 2.13) | 0.29 |
| Monocyte | 22.78 (4.40 - 118.10) | <0.001 | 22.78 (4.40 - 118.10) | <0.001 |
| Neutrophil (%) | 1.06 (1.01 - 1.12) | 0.02 | 1.06 (1.01 - 1.12) | 0.02 |
| Lymphocyte (%) | 0.90 (0.84 - 0.97) | 0.004 | 0.90 (0.84 - 0.97) | 0.004 |
| Monocyte (%) | 0.98 (0.81 - 1.19) | 0.86 | 0.98 (0.81 - 1.19) | 0.86 |
| Red blood cell count | 5.41 (2.40 - 12.20) | <0.001 | 5.41 (2.40 - 12.20) | <0.001 |
| Hemoglobin | 1.07 (1.04 - 1.10) | <0.001 | 1.07 (1.04 - 1.10) | <0.001 |
| Hematocrit | 1.26 (1.15 - 1.39) | <0.001 | 1.26 (1.15 - 1.39) | <0.001 |
| Red Cell Distribution Width - Coefficient of Variation (%) | 1.30 (0.90 - 1.89) | 0.17 | 1.30 (0.90 - 1.89) | 0.17 |
| Red cell distribution width - standard deviation | 1.22 (1.08 - 1.38) | 0.002 | 1.22 (1.08 - 1.38) | 0.002 |
| Platelet count | 1.00 (0.99 - 1.00) | 0.55 | 1.00 (0.99 - 1.00) | 0.55 |
| Neutrophil to lymphocyte and platelet ratio | 1.17 (1.07 - 1.28) | <0.001 | 1.17 (1.07 - 1.28) | <0.001 |
| Mean platelet volume | 1.16 (0.93 - 1.45) | 0.18 | 1.16 (0.93 - 1.45) | 0.18 |
| Plateletocrit | 1.20 (0.01 - 288.04) | 0.95 | 1.20 (0.01 - 288.04) | 0.95 |
| Platelet Large Cell Count | 1.01 (0.99 - 1.03) | 0.24 | 1.01 (0.99 - 1.03) | 0.24 |
| Platelet Large Cell Ratio | 1.02 (0.99 - 1.05) | 0.22 | 1.02 (0.99 - 1.05) | 0.22 |
| Neutrophil to lymphocyte ratio | 1.07 (1.03 - 1.11) | <0.001 | 1.07 (1.03 - 1.11) | <0.001 |
| Monocyte-to-Lymphocyte Ratio | 6.78 (2.24 - 20.52) | <0.001 | 6.78 (2.24 - 20.52) | <0.001 |
| Prothrombin time | 0.50 (0.34 - 0.74) | <0.001 | 0.52 (0.36 - 0.76) | <0.001 |
| International normalized ratio | 0.00 (0.00 - 0.03) | <0.001 | 0.00 (0.00 - 0.04) | <0.001 |
| Activated partial thromboplastin time | 0.99 (0.97 - 1.01) | 0.33 | 0.99 (0.97 - 1.01) | 0.33 |
| Thrombin time | 0.99 (0.98 - 1.01) | 0.55 | 0.99 (0.98 - 1.01) | 0.41 |
| Fibrinogen | 1.72 (1.01 - 2.95) | 0.050 | 1.73 (1.01 - 2.97) | 0.048 |
| Total bilirubin | 1.03 (0.99 - 1.07) | 0.18 | 1.02 (0.98 - 1.06) | 0.30 |
| Albumin | 1.02 (0.95 - 1.10) | 0.54 | 1.03 (0.96 - 1.10) | 0.43 |
| Globulin | 1.07 (1.01 - 1.15) | 0.037 | 1.08 (1.01 - 1.15) | 0.030 |
| Albumin-globulin ratio | 0.37 (0.09 - 1.61) | 0.19 | 0.35 (0.08 - 1.52) | 0.16 |
| 1/Albumin-globulin ratio | 7.17 (0.43 - 120.57) | 0.17 | 6.91 (0.41 - 115.56) | 0.18 |
| Alanine aminotransferase | 1.04 (1.02 - 1.06) | 0.001 | 1.04 (1.02 - 1.06) | 0.001 |
| Aspartate aminotransferase | 1.07 (1.04 - 1.11) | <0.001 | 1.07 (1.04 - 1.11) | <0.001 |
| Total bilirubin | 1.03 (0.99 - 1.08) | 0.16 | 1.03 (0.99 - 1.07) | 0.20 |
| Direct bilirubin | 1.02 (0.92 - 1.13) | 0.73 | 1.01 (0.92 - 1.12) | 0.81 |
| Indirect Bilirubin | 1.02 (0.98 - 1.07) | 0.36 | 1.02 (0.97 - 1.06) | 0.46 |
| γ-glutamyl transpeptidase | 1.02 (1.01 - 1.04) | 0.025 | 1.02 (1.01 - 1.04) | 0.027 |
| Alkaline phosphatase | 1.00 (1.00 - 1.01) | 0.33 | 1.01 (1.00 - 1.01) | 0.29 |
| Cholinesterase | 1.01 (1.01 - 1.01) | 0.005 | 1.01 (1.01 - 1.01) | 0.005 |
| Creatine kinase | 1.01 (1.01 - 1.01) | 0.018 | 1.01 (1.01 - 1.01) | 0.014 |
| Creatine kinase-MB Mass | 1.24 (1.06 - 1.45) | 0.007 | 1.18 (1.05 - 1.33) | 0.006 |
| Carbon dioxide combining power | 0.79 (0.71 - 0.89) | <0.001 | 0.79 (0.72 - 0.88) | <0.001 |
| Blood urea nitrogen | 1.63 (1.29 - 2.07) | <0.001 | 1.62 (1.28 - 2.04) | <0.001 |
| Serum creatinine | 1.04 (1.02 - 1.06) | <0.001 | 1.04 (1.02 - 1.06) | <0.001 |
| Estimated Glomerular filtration rate | 0.96 (0.94 - 0.97) | <0.001 | 0.96 (0.94 - 0.97) | <0.001 |
| Lactate dehydrogenase | 1.01 (1.01 - 1.02) | 0.002 | 1.01 (1.01 - 1.02) | <0.001 |
| Na^+^ | 0.96 (0.86 - 1.07) | 0.44 | 0.98 (0.89 - 1.08) | 0.69 |
| K^+^ | 0.77 (0.35 - 1.68) | 0.51 | 0.82 (0.40 - 1.69) | 0.59 |
| Cl^-^ | 1.03 (0.94 - 1.12) | 0.53 | 1.01 (0.93 - 1.10) | 0.77 |
| Ca^2+^ | 1.07 (0.50 - 2.31) | 0.86 | 1.23 (0.61 - 2.51) | 0.56 |
| Gender |  |  |  |  |
| male | 1.00 (Reference) |  | 1.00 (Reference) |  |
| female | 0.35 (0.16 - 0.79) | 0.011 | 0.35 (0.16 - 0.79) | 0.011 |
| Blood pressure |  |  |  |  |
| Normal | 1.00 (Reference) |  | 1.00 (Reference) |  |
| Hypertension | 19.73 (5.71 - 68.16) | <0.001 | 19.73 (5.71 - 68.16) | <0.001 |
| Hypotension | 61.38 (7.40 - 509.51) | <0.001 | 61.38 (7.40 - 509.51) | <0.001 |
| Respiration |  |  |  |  |
| Normal | 1.00 (Reference) |  | 1.00 (Reference) |  |
| Tachypnea | 10.61 (2.26 - 49.73) | 0.003 | 10.61 (2.26 - 49.73) | 0.003 |
| Slowing | 1.80 (0.13 - 24.16) | 0.66 | 1.80 (0.13 - 24.16) | 0.66 |
| Slow down after a hurry | 7.36 (1.34 - 40.54) | 0.022 | 7.36 (1.34 - 40.54) | 0.022 |
| Heart |  |  |  |  |
| Normal | 1.00 (Reference) |  | 1.00 (Reference) |  |
| High heart rate | 17.14 (5.81 - 50.56) | <0.001 | 17.14 (5.81 - 50.56) | <0.001 |
| Low heart rate | 14.29 (1.14 - 178.87) | 0.039 | 14.29 (1.14 - 178.87) | 0.039 |
| High first and then low | 85.71 (9.61 - 764.32) | <0.001 | 85.71 (9.61 - 764.32) | <0.001 |
| Fever |  |  |  |  |
| No | 1.00 (Reference) |  | 1.00 (Reference) |  |
| Yes | 0.81 (0.18 - 3.60) | 0.79 | 0.81 (0.18 - 3.60) | 0.79 |
| Bowel sounds |  |  |  |  |
| Normal | 1.00 (Reference) |  | 1.00 (Reference) |  |
| Weakening | 26607313.34 (0.00 - Inf) | 0.99 | 26607313.34 (0.00 - Inf) | 0.99 |
| Active | 1.39 (0.52 - 3.74) | 0.51 | 1.39 (0.52 - 3.74) | 0.51 |
| Lung rales |  |  |  |  |
| No | 1.00 (Reference) |  | 1.00 (Reference) |  |
| Yes | 19.74 (2.44 - 159.37) | 0.005 | 19.74 (2.44 - 159.37) | 0.005 |
| Nausea and Vomiting |  |  |  |  |
| No | 1.00 (Reference) |  | 1.00 (Reference) |  |
| Yes | 4.53 (1.42 - 14.45) | 0.011 | 4.53 (1.42 - 14.45) | 0.011 |
| Abdominal pain |  |  |  |  |
| No | 1.00 (Reference) |  | 1.00 (Reference) |  |
| Yes | 1.71 (0.78 - 3.75) | 0.18 | 1.71 (0.78 - 3.75) | 0.18 |
| Diarrhea |  |  |  |  |
| No | 1.00 (Reference) |  | 1.00 (Reference) |  |
| Yes | 1.35 (0.53 - 3.41) | 0.53 | 1.35 (0.53 - 3.41) | 0.53 |
| Oliguria and Anuria |  |  |  |  |
| No | 1.00 (Reference) |  | 1.00 (Reference) |  |
| Yes | 4.71 (1.88 - 11.81) | <0.001 | 4.71 (1.88 - 11.81) | <0.001 |
| Dysphoria |  |  |  |  |
| No | 1.00 (Reference) |  | 1.00 (Reference) |  |
| Yes | 9.97 (3.37 - 29.54) | <0.001 | 9.97 (3.37 - 29.54) | <0.001 |
| Delirium |  |  |  |  |
| No | 1.00 (Reference) |  | 1.00 (Reference) |  |
| Yes | 13.19 (1.59 - 109.72) | 0.017 | 13.19 (1.59 - 109.72) | 0.017 |
| Convulsions |  |  |  |  |
| No | 1.00 (Reference) |  | 1.00 (Reference) |  |
| Yes | 73271621.26 (0.00 - Inf) | 0.99 | 73271621.26 (0.00 - Inf) | 0.99 |
| Comatose |  |  |  |  |
| No | 1.00 (Reference) |  | 1.00 (Reference) |  |
| Yes | 82430573.94 (0.00 - Inf) | 0.99 | 82430573.94 (0.00 - Inf) | 0.99 |
| Mental confusion |  |  |  |  |
| No | 1.00 (Reference) |  | 1.00 (Reference) |  |
| Yes | 40.67 (5.16 - 320.33) | <0.001 | 40.67 (5.16 - 320.33) | <0.001 |
| Dyspnea |  |  |  |  |
| No | 1.00 (Reference) |  | 1.00 (Reference) |  |
| Yes | 30.50 (3.85 - 241.92) | 0.001 | 30.50 (3.85 - 241.92) | 0.001 |
| Muscular pain |  |  |  |  |
| No | 1.00 (Reference) |  | 1.00 (Reference) |  |
| Yes | 3.03 (0.71 - 12.82) | 0.13 | 3.03 (0.71 - 12.82) | 0.13 |

OR, odds ratio; CI, Confidence Interval.

**Table 4: Variable definition table and Variable unit**

| Variables | measure | threshold |  |
| --- | --- | --- | --- |
| Blood pressure | mmHg | normal |  |
|  |  | Hypertension | ≥140/90 |
|  |  | Hypotension | ＜90/60 |
| Respiration | Times/min | normal |  |
|  |  | Tachypnea | ≥24 |
|  |  | Slowing | ＜12 |
|  |  | Slow down after a hurry | From ≥24 to ＜12 |
| Heart rate | Times/min | normal |  |
|  |  | High heart rate | ＞100 |
|  |  | Low heart rate | ＜60 |
|  |  | High first and then low | From ＞100 to ＜60 |
| Fever | ℃（armpit） | No |  |
|  |  | Yes | ≥37.3 |
| Bowel sounds | Times/min | normal |  |
|  |  | Weakening | <4 |
|  |  | active | >6 |
| Oliguria and Anuria | mL/24h | No |  |
|  |  | Yes | <400 |
| White blood cell count | 10^9^/L |  |  |
| Neutrophil | 10^9^/L |  |  |
| Lymphocyte | 10^9^/L |  |  |
| Monocyte | 10^9^/L |  |  |
| Red blood cell count | 10^12^/L |  |  |
| Hemoglobin | g/L |  |  |
| Hematocrit | % |  |  |
| Red Cell Distribution Width-Coefficient of Variation (%) | % |  |  |
| Red cell distribution width-standard deviation | fL |  |  |
| Platelet count | 10^9^/L |  |  |
| Neutrophil to lymphocyte and platelet ratio | % |  |  |
| Mean platelet volume | fL |  |  |
| Plateletocrit | % |  |  |
| Platelet Large Cell Count | 10^9^/L |  |  |
| Platelet Large Cell Ratio | % |  |  |
| Prothrombin time | s |  |  |
| Activated partial thromboplastin time | s |  |  |
| Thrombin time | s |  |  |
| Fibrinogen | g/L |  |  |
| Total bilirubin | g/L |  |  |
| Albumin | g/L |  |  |
| Globulin | g/L |  |  |
| Alanine aminotransferase | U/L |  |  |
| Aspartate aminotransferase | U/L |  |  |
| Total bilirubin | umol/L |  |  |
| Direct bilirubin | umol/L |  |  |
| Indirect Bilirubin | umol/L |  |  |
| γ-glutamyl transpeptidase | U/L |  |  |
| Alkaline phosphatase | U/L |  |  |
| Carbon dioxide combining power | mmol/L |  |  |
| Estimated Glomerular filtration rate | ml/min |  |  |
| Blood urea nitrogen | mmol/L |  |  |
| Serum creatinine | umol/L |  |  |
| Cholinesterase | U/L |  |  |
| Creatine kinase | U/L |  |  |
| Creatine kinase-MB Mass | ng/mL |  |  |
| Lactate dehydrogenase | mmol/L |  |  |
| Na^+^ | mmol/L |  |  |
| K^+^ | mmol/L |  |  |
| Cl^-^ | mmol/L |  |  |
| Ca^2+^ | mmol/L |  |  |
| plasma DQ concentration | ng/mL |  |  |
